# Investigation of the dehydration of ulexite ore with different parameters and modeling with artificial neural network (ANN) method

**DOI:** 10.55730/1300-0527.3531

**Published:** 2022-12-07

**Authors:** Mustafa Engin KOCADAĞİSTAN

**Affiliations:** Department of Metallurgical and Materials Engineering, Faculty of Engineering, Atatürk University, Erzurum, Turkey

**Keywords:** Ulexite, dehydration, ANN, modeling, heat treatment

## Abstract

Study experiments were conducted considering the temperature, time, and sample weight parameters in order to model the dehydration by applying dehydration processes to ulexite ores. Data obtained from the dehydration processes of ulexite ore were compared with TG analyzes. It was observed as the result of the heat treatment that the fastest water removal was provided in the temperature range of 150–250 °C and it was very low in the range of 400–750 °C. In order to design the ANN method, 4 different models were proposed with the same parameters in the dehydration experiments and the network structure was determined. The performance of the ANN model was assessed by means of error measurements i.e. absolute error (AE), absolute relative error (ARE) and coefficient of determination (R^2^). The mean value of R^2^ was 99%. It was found that the independent variables explained the dependent variable efficiently and the models were very successful. It was shown that the new models can be created using genetic algorithms or hybrid methods in the future studies requiring fewer experiments by following the same process in the present study.

## 1.Introduction

Boron ore, which is commonly known as borates and borosilicates, and always existent in the nature as a function of oxygen, ranks the 51st among the elements [1]. It contains different amounts of B_2_O_3_ in its structure, gains value and expands itsapplication fields depending on the ratio of B_2_O_3_ and H_2_O (crystalline water) [2–5]. Among the most important ores forthe boron industry are borax, colemanite, ulexite and kernite. In general, ulexites occur in the form of soft, very moist andfibrous crystals. The hardness and specific gravity of ulexite, which contains 42%–43% B_2_O_3_ in its pure form, is 2.5 and1.955 g/cm^3^. Its chemical composition is Na_2_O.2CaO.5B_2_O_3_.16 H_2_O. Borax, halite (NaCl), colemanite and other combinedminerals such as calcium borate are formed from ulexite [6]. Ulexite ore is mined mainly in Turkey (Kırka, Bigadiç, andEmet deposits), at Lake Inder in Kazakhstan and in Argentina [7].

The heat treatments applied to boron ores are commonly known as dehydration or calcination/dehydroxylation [3–5]. In these heat treatments, chemically bound water to the minerals is removed and the minerals undergo structural changes [8]. Calcination removes not only water but also carbon dioxide or other chemically bound gases such as hydrates and carbonates. Some studies in the literature on differential thermal analysis and calcination processes of boron minerals resulted in patents in the world [9–11]. The dehydration process can remove much of the crystalline water in ulexite as a function of temperature [12]. Dehydration of ulexite ores begins in the range of 50–100 °C by reaching its maximum rate in the range of 230–250 °C. Between 400 °C and 500 °C, the dehydration rate is very low and almost no weight loss is observed at higher temperatures. Şener et al. (2000) found in their study that amorphization occurs above 160 °C [5].

It was found in a study conducted on the dehydration of boron minerals that all hydrated boron minerals get rid of their crystallization water partly or wholly at certain temperatures and undergo various structural changes. On the other hand, some boron minerals such as ulexite gradually release their crystal water during calcination and transform into a more porous structure without decomposing [13]. It was found in another study conducted by Özbayoğlu where the calcination properties of ulexite and colemanite ores were investigated that the mixed minerals were calcined at 450 °C for 30 min and colemanite decrepitates and ulexite transforms into a hard structure [14,15]. In another study on the decrepitation of colemanite+ ulexite minerals, the optimum decrepitation conditions for colemanite were set at 15 min at 500 °C, and it was found that grain size is not important in the range of 0.5–19 mm. Although ulexite is not decrepit, its structure was observed to collapse [3]. Hoskan (2019) studied the calcinations of boron ores and the separation of undesirable minerals such as clay and calcite from calcined boron minerals [16]. The highest B_2_O_3_ content of colemanite ore was found to be 55.25% with 80.82% recovery at 600 °C in 30 min. For ulexite ore, the highest B_2_O_3_ content was found to be 53.35% and 35.89% recovery in 60 min and at 600 °C.

Dehydration of hydrated boron minerals has been studied for a long time [17,18]. Besides, there is a different use of boron ores in industry. For example, Yanen et al. (2022) investigated the thermophysical properties of colemanite, ulexite, and tincal reinforced polyester composites in their study and they found that the thermal stability of the polyester composite was increased with the addition of boron ores [19].

Thermogravimetric (TG) methods are used to study the kinetics of many decomposition reactions in the solid state. Thermogravimetric curves are a function of reaction kinetics and the data obtained from these curves eases the evaluation of kinetic parameters [20].

Artificial neural networks (ANN) are used as a data modeling tool and provide good results [21]. ANNs were developed to explore the science of neurobiology and apply the knowledge gained to computer science. ANNs are a class of flexible nonlinear models that can adaptively find patterns from data. ANN is a type of computing paradigm inspired by the human brain. The structure of the computer system is considered as the key element of this paradigm [22]. A large number of connections are made between processing elements to solve certain problems in harmony. ANNs learn and are trained by examples like human intelligence. Training occurs with some acceptable error. The advantage of ANNs over other models is their ability to model a multivariable problem given the complex relationships between variables. Moreover, ANNs can extract the implicit nonlinear relationships between these variables by “learning” with training data [23].

Although many different types of models have been proposed for modeling, the most popular model in such applications is the feedforward network type. In the model ANN, the independent variables are referred to as inputs and the dependent variable is referred to as output [23–25]. The input parameter severity table shows the conceptual significance of each input column. The significance of the input column is calculated as the decrease in network performance after the input is removed and not used by the network [26]. ANNs consist of many nodes connected by synapses working in parallel. ANNs consist of many nodes working in parallel, all connected by linking synapses. The greatest advantage of a neural network is its ability to model a complex nonlinear relationship without having to make prior assumptions about the nature of the relationship as in a block diagram [27]. In other words, ANNs overcome the limitations of the traditional approach by extracting the desired information directly from the data. ANNs are commonly used in models related to estimation studies. The main advantages of ANNs are that they are fault tolerant, can work with missing data, and use the examples in learning processes [25].

In this context, the ANN method and the studies on the subject were studied and it was shown that it is used in many areas of our daily life. In a study by Thiago da Silva Ribeiro et al. (2019), boron removal from mine wastewater was carried out using the electrocoagulation method and ANN modeling was performed with the data obtained. The model with 3-10-1 topology gave satisfactory results in determining the optimal conditions. The square of error, the sum of 0.616 and the correlation coefficient (R^2^) were found to be 0.973, confirming that the model ANN is compatible with these results [28]. In another study, the dissolution of colemanite ore in water solutions saturated with CO_2_ was investigated. For this purpose, the dissolution rate was estimated using multilayer perceptron-based artificial neural networks (ANN). ANN provided very accurate predictions compared to a mathematical model built using the regression method. It was shown that ANNs can be preferred instead of traditional statistical methods [29].

Production of waste polyethylene terephthalate reinforced biocomposite with RSM design and evaluation of thermophysical properties with ANN were carried out. The density, Shore D hardness, elastic modulus, thermal conductivity, and thermal stability of the palm oil-based BUP composite were evaluated both experimentally and by RSM and ANN simulations. It was found that the desired properties of the obtained biocomposite according to the purpose of use can be designed according to the RSM optimization [30].

In this study, by applying the dehydration process to ulexite ore; calculating the percent weight reductions versus the weight losses obtained as a function of temperature, ore amount, and time; and disclosing the dehydration process of ulexite, comparison with dehydration by TG analysis and the modelability of the dehydration process by the ANN method were investigated. In addition, by determining the changes in the amounts of B_2_O_3_ and other oxidized compounds, the dehydration of ulexite ore was characterized, the changes due to heat treatments were observed, and the importance of dehydration in mineral enrichment was highlighted. The most important feature of this study is that the predictive power of the model, resulting from the inclusion of data obtained from the dehydration processes is very high.

## 2. Materials and methods

### 2.1. Materials

**Figure 1:**
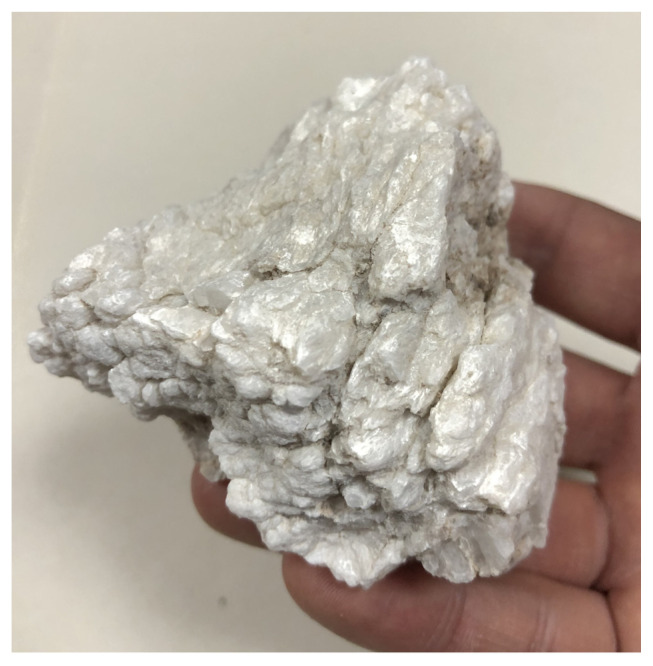
Ulexite-run ore sample.

The ore samples were obtained from the General Directorate of Mining in ETI, Bigadiç Boron Operations Directorate (Figure 1). The content of the ore is 43.1% B_2_O_3_. The chemical composition of the ore was determined by the XPS method at Atatürk University East Anatolia High Technology Research and Application Center (DAYTAM). Using the data obtained as a result of XPS, the amounts of B_2_O_3_, Na_2_O, and CaO in the composition of the ulexite ore were compared with the stoichiometrically calculated amounts shown in Table 1. For the calcination/dehydration experiments, the ulexite ore was subjected to sample preparation. First, it was crushed in a jaw crusher to a mesh size of 60 and then ground in a ball mill to a size of 325 mesh. The ore to be used for the experiments was then screened with a mechanical sieve to obtain the final product with a size of −325 mesh. The furnace used for the calcination/dehydration experiments was a Nabertherm brand muffle furnace with a maximum temperature of 1100 °C. TG Analyses were carried out in the Department of Chemical Engineering, Faculty of Engineering, Atatürk University, on a Netzsch STA 409 PC LUXX model TG instrument. ANN was used for modelling and the Levenberg-Marquardt algorithm was chosen as the training algorithm in ANN models.

## 2.2 Methods

### 2.2.1 Experiments on dehydration

Experiments on dehydration or thermal analysis were conducted using two methods, static and dynamic. In the experiments conducted using the static method, specific amounts of samples were held at specific temperatures and times. The heating process was carried on until a constant weight was reached. Then the cooled samples were weighed and the changes in weight due to dehydration or decomposition of the samples at specific temperatures were determined [31,32]. Various parameters for the removal of chemically bound water in the ore were selected during the experiments. The parameters to be used in this study were dehydration temperature and time and the amount of ore, which are listed in Table 2. Ceramic crucibles without lids in cylindrical shape with a volume of 35 mL were used for the experiments. In the procedure, the furnace was first set to the desired temperature with the empty crucible and then the experiment was started after the sample was immediately placed in the preheated porcelain crucible. At the end of each experiment, the calcined samples were allowed to cool down to room temperature in a desiccator to avoid moisture absorption. For the calcination/dehydration experiments, the oven was initially set at 50 °C. When the oven reached this temperature, the ulexite samples weighing 1, 3, and 5 g, which were weighed on a precision balance, were placed in porcelain crucibles and placed in the oven. The process was started for the periods of 1, 2, 4, and 6 h specified as calcination/dehydration time. After 1 h, 1, 3, and 5-gram samples were removed from the oven while the oven temperature was 50 °C. Then, at the end of the 2nd, 4th, and 6th hour, the same procedures were repeated and the experiments were completed at the selected dehydration temperatures. Each sample removed from the oven was first cooled in a desiccator and then weighed and the weight differences were determined.

### 2.2.2. TG analysis

Thermogravimetric analysis (TG) is a technique that records the changes in a sample mass as a function of temperature [33]. In this method, the decrease in the mass of the analyzed substance as a result of programmatically increased or decreased temperature is studied as a function of temperature or time. Together with the preliminary ore, 3 heat-treated samples were selected considering the parameters of time period (2 h), amount (1 g) and temperature (200 °C, 350 °C, and 400 °C) and TG analyzes were performed on a total of 4 different samples. Analyzes were performed using an aluminum oxide crucible at a heating rate of 10 °C/min at room temperature and at 700 °C in air. This process was performed to compare and verify the data obtained during dehydration and modeling with the TG analyzes.

### 2.2.3 Modeling with ANN

Artificial neural networks (ANN) are generally used for the prediction, classification, association, interpretation and filtering of data [34,26]. ANN is widely used in many application areas, such as pattern recognition, system diagnosis, robotics, signal processing and nonlinear control domains [35,36]. Technically, the task of the ANN is to produce an output in response to a given set of data. For this purpose, the network is trained with certain examples. Then the network reaches a level where it can generalize and make decisions. With this acquired ability, the outputs of the network are then determined.

Before applying the method ANN, all factors related to the dehydration processes for ulexite and other boron ores are evaluated. As a result of this evaluation, the main parameters affecting dehydration are determined. The ANN method was preferred because of the great number of effective factors and the complex relationships between them. The number of studies in the related literature on the optimization and modeling of the thermal processes using ANN is not sufficient besides the dehydration/calcination processes applied to boron ores. It is an important innovation that methods such as ANN are used to obtain boron ore with the desired amount of water and B_2_O_3_ content without applying the dehydration process. The importance of such methods is greater for industrial applications, where the demand for boron ores with different B_2_O_3_ and water contents is high.

**Figure 2:**
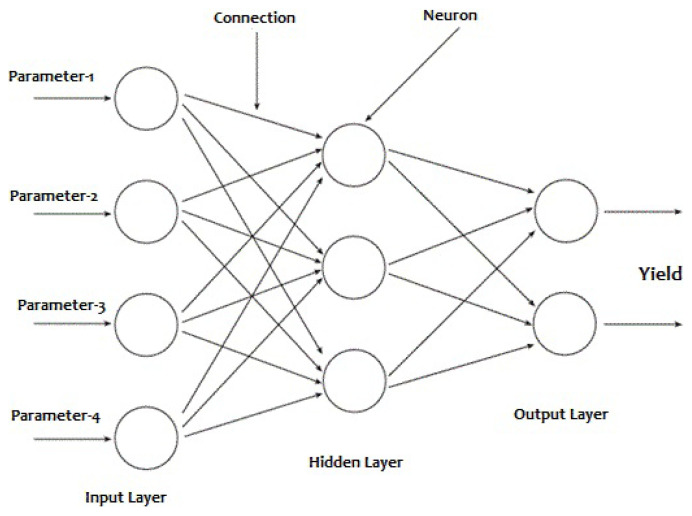
Neural network structure.

In this study, candidate parameters for the model ANN were selected and data were collected by dehydration experiments. Before constructing a neural network model for the data obtained from dehydration, dimensionless groups must be selected to be used as input and target parameters. Then, the neural network structure was created and the input, hidden and output layers were determined (Figure 2). Input layers are the terminals where input data sets are presented to the network. The number of neurons in this layer is equal to the number of input data. Each input neuron receives one data. Hidden layer is the layer that performs the main function of the network. The number of hidden layers and neurons varies according to the problem and is completely under the control of the network designer. This layer processes the weighted data received from the input layer with a function suitable for the problem and transmits it to the next layer. The output layer is the outermost layer of the network. It processes the data it receives from the hidden layer with the function used by the network and outputs it. The number of neurons in the output layer is equal to the number of outputs of each data presented to the network. The values obtained from this layer are the output values of the ANN for the problem in question [37].

**Figure 3:**
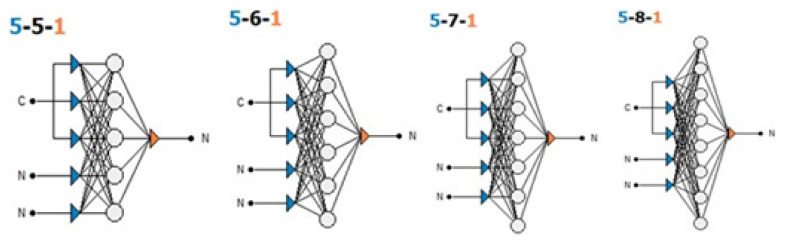
Network structure models: [model-1 for B_2_O_3_ (5-5-1), model-2 for CaO (5-6-1), model-3 for H_2_O (5-7-1) and model-4 for Na_2_O (5-8-1)] and (C = dehydration temperature-ºC, N = dehydration time-h and amount of ore-g)].

The Levenberg-Marquardt algorithm was used as a training algorithm in ANN models in the study. The input variables for the different models were determined considering the variables and correlation matrix used in previous studies. In the preprocessing of the network, both the input and output variables were normalized in the range of 0–1 using a minimax algorithm. Table 2 shows the parameters used in the creation of the ANN models. Then, the best networks were selected in the design section (Figure 3).

Four models were created with the parameters used (Figure 3). For the activation function in the hidden layer, the network structures (model-1 (B_2_O_3_): 5-5-1, model-2 (CaO): 5-6-1, model-3 (H_2_O): 5-7-1, and model-4 (Na_2_O): 5-8-1) were determined. In the next step, the training of the network was started using training algorithms. In this phase, the importance of the parameters and the training algorithm were created. The training procedure is the most important part of ANN modelling. In the developed models, 70% of the data were used as training data, 15% as test data and 15% as validation data. According to Equation 1, the expected increase in the amount of Y, B_2_O_3_, Na_2_O, and CaO and the expected decrease in the amount of H_2_O are the weighting matrices W1 and W2, and b1 and b2 are the vectoral deviations.


(1) $$Y=f_{2}\;\left\{\left[W_{2}]f_{1}\;\left[\left[W_{1}]\;\left[\begin{array}{c} dehydration\;temperature\\ dehydration\;time\\ amount\;of\;ore\\ ore\;grain\;size \end{array}\right]\;[+b_{1}\right]\right]\;[+b_{2}\right]\right\}$$

The efficiency of the backpropagation training algorithm depends on the number of neurons in the hidden layer. Different numbers of neurons in the hidden layer from 1 to 29 were tested. The equations used in calculating the model error are shown below (Equations 2–4).


(2) $$AE=\left|E_{obs}-E_{pre}\right|$$

(3) $$ARE=\left|\frac{E_{obs}-E_{pre}}{E_{obs}}\right|$$

(4) $$R^{2}=1-\left(\sum_{i-1}^{n}AE^{2}\right)/\left(\sum_{i-1}^{n}{\left(E_{obs}-\bar{E_{obs}}\right)}^{2}\right)$$

### 3. Results and discussion

**Figure 4:**
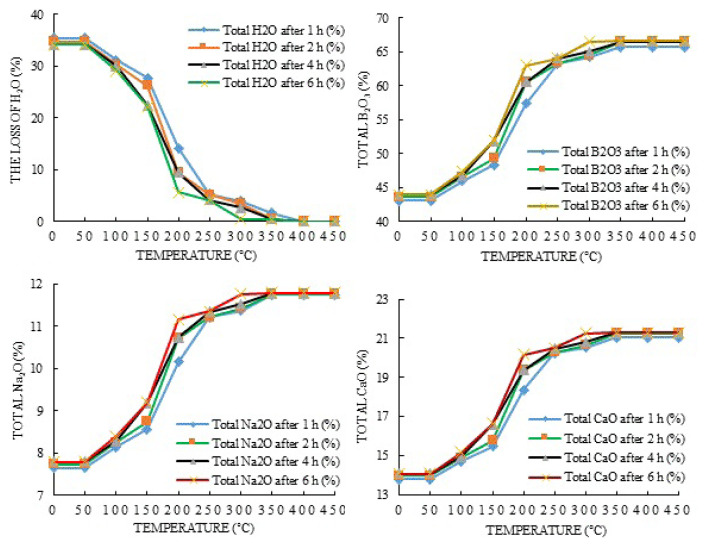
The changes in B_2_O_3_, Na_2_O, and CaO contents of ulexite ore (1 g) as a result of H_2_O removal during dehydration processes.

**Figure 5:**
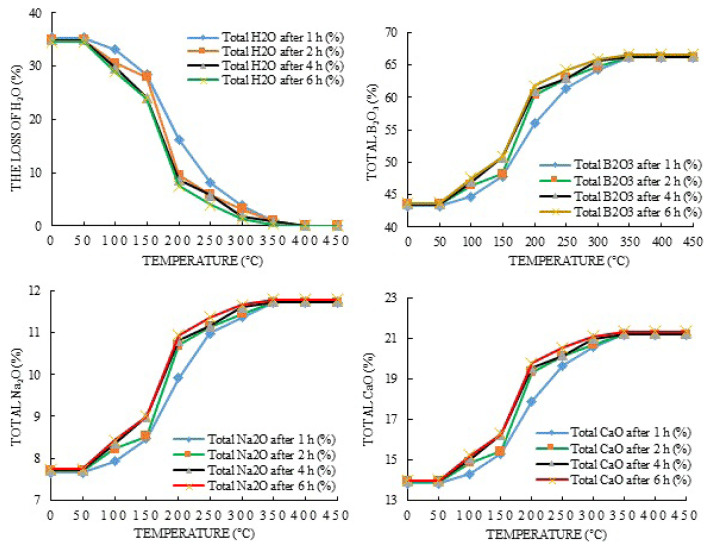
The changes in B_2_O_3_, Na_2_O, and CaO contents of ulexite ore (3 g) as a result of H_2_O removal during dehydration processes.

After the completion of the dehydration experiments, weight losses were calculated according to the amount of ore. It was observed from the calculations that while the content of H_2_O decreased, the amount of B_2_O_3_, Na_2_O, and CaO increased. The plots of these values are shown in Figures 3–5.

**Figure 6:**
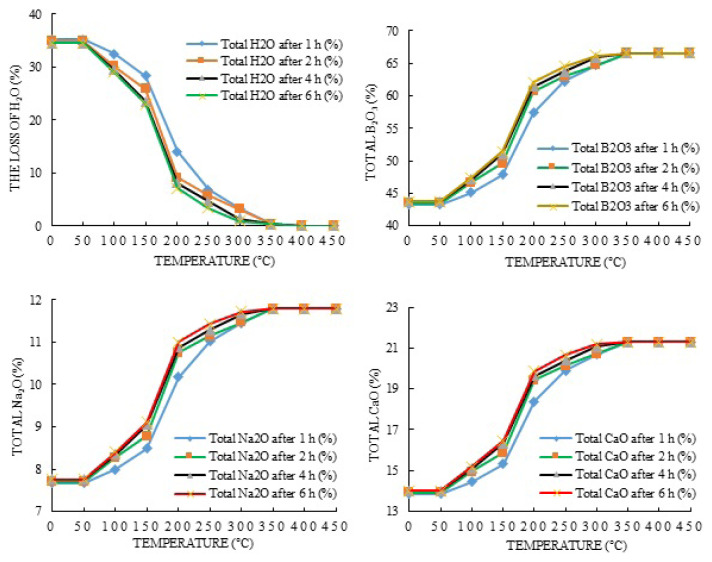
The changes in B_2_O_3_, Na_2_O, and CaO contents of ulexite ore (5 g) as a result of H_2_O removal during dehydration processes.

It is seen when considered Figures 3–5 for the changes in the amounts of H_2_O that they begin to decrease with 1, 3, and 5-g ulexite ore contents in the temperature range of 50–100 °C. The increase in water loss accelerates in the temperature range of 150–200 °C and reaches up to the maximum level in the temperature range of 200–350 °C. Beyond 400 °C, the decrease in the amount of H_2_O becomes stable and almost all (99.5%) water in the ore is removed. It can be seen that the decrease in weight that occurs in dehydration processes after 50 °C corresponds to the release of water in the physical sense. In the dehydration processes, at the end of the 1-h period and in the temperature range of 350–400 °C for 1-, 3-, and 5-g ore samples, the amount of water remaining in the ore was determined to be 1.57%, 0.94%, and 0.38%, respectively. At the end of the 2-h period and in the temperature range of 350–400 °C, the water content was found to be about 0.49%, 0.91%, and 0.37% for 1-, 3-, and 5-g ore samples, respectively. At the end of the 4-h period and in the temperature range of 350–400 °C for 1-, 3-, and 5-g ore samples, the amount of water remaining in the ore was found to be 0.44%, 0.78%, and 0.36%, respectively. At the end of the 6-h period and in the temperature range of 350–400 °C, water content was found to be 0.33%, 0.26%, and 0.33% for 1-, 3-, and 5-g ore samples, respectively. Based on the findings, it can be seen that during dehydration, the highest water removal efficiency was achieved at the end of 6 h, but beyond 400 °C, the amount of H_2_O in the ore (for each time and amount of ore) was almost the same and completely removed. Thus, the total weight loss that occurred during the dehydration studies of ulexite ore was about 35%. Stoichiometric calculations indicate that boric acid may contain 34%–35% hydrated water. “Rapid” weight reduction occurred between 150 °C and 200 °C, “slowing” of weight reduction was observed after 200 °C, and partial “stability” occurred after 350–400 °C. This situation can be clearly seen in Figures 4–6. While the rate of dehydration increases significantly up to 300 °C, the weight loss slows down from 350 °C, and the weight loss due to dehydration is almost complete at about 400 °C.

**Figure 7:**
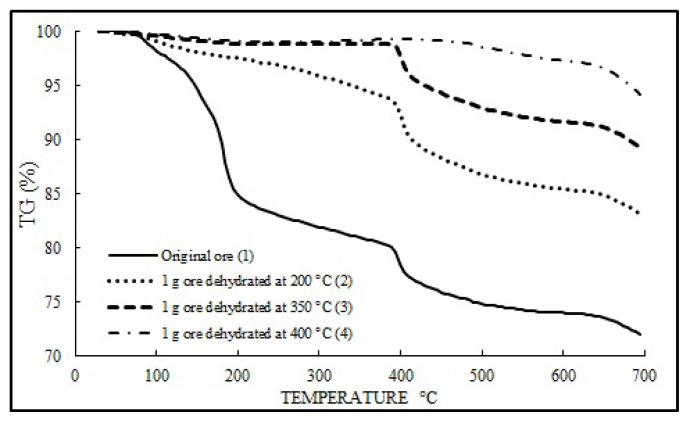
TG analysis results of run-of-the-mill ore and ores dehydrated at 200, 300, and 400 °C.

Graphs representing the results of the thermal analysis (TG) performed to study the dehydration of ulexite can be found in Figure 7. Looking at the TG curve of the differential thermal analysis of ulexite, it was found that the weight loss at 400 °C is in the range of 34%–35%. In the TG curve, the weight decrease that started at 50–100 °C continues until 400–450 °C, and then the curve becomes stable. This situation is consistent with the values found between the same temperatures in the dehydration curves. Most of this weight loss occurred between 50 °C and 350 °C and corresponds to 99% of the total weight loss. Figure 7 shows that the maximum yield (99.5%) is reached after 6 h for the amounts of 1-, 3-, and 5-g H_2_O. Similarly, the lowest water removal (95.5%) was obtained for 1 h and 1-g ore. The amount of water remaining in sample 2 during the dehydration processes was found to be 9.42% theoretically, which accounts for approximately 90%–95% and is consistent with the TG graph. Likewise, the amount of water remaining in the 3rd sample was found to be 0.49% theoretically, again confirming the water loss of around 99% when compared with the TG graph. The same trend is valid for sample 4. This means that if TG is applied to an ore sample with 10% water content, curve 2 in the graph will be obtained. Figure 7 shows that the maximum yield (99.5%) is reached after 6 h for 1-, 3-, and 5-g H_2_O. Similarly, when examining the samples of 1-g ore at temperatures of 200 °C and 350 °C, it is clear that the rate of the remaining water in the ore is about 5%. These analyzes show that there is a complete agreement between the dehydration processes and the TG analyzes, and accurate data were obtained for ANN modeling.

Accordingly, the dehydration of ulexite ore was studied at temperatures between 50 °C and 750 °C and it appears from the results obtained that 99.5% of the H_2_O content in its body is removed until reaching 400 °C. As a result of the dehydration processes, it was found that the water removal rates calculated by weight differences are in exact agreement with the data obtained from TG. The water in the ore was found to decrease slowly up to 150 °C, accelerate in the range 150–250 °C, and slowdown in the range of 250–400 °C by leaving all the water behind.

**Figure 8:**
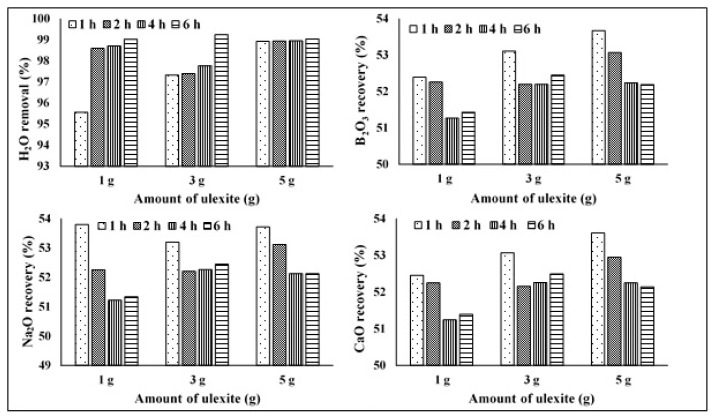
Change rates of H_2_O, B_2_O_3_, Na_2_O and CaO as a result of dehydration processes (%).

The changes in the contents of B_2_O_3_, CaO, and Na_2_O in the ore at the end of the dehydration processes were also studied. Up to a temperature of 150 °C, the increase in the contents of B_2_O_3_, CaO, and Na_2_O is small. It is observed that this increase accelerates in the range of 150–250 °C and slows down again in the range of 250–400 °C. At the end of the experiments, the B_2_O_3_ content was between 66% and 67%, the CaO content was between 21% and 22%, and the Na_2_O content was 11%–12% (Figures 3–5). The content changes were stable in the range of 350–400 °C and exhibited changes of the order of 0.001% up to 750 °C. That is, although the changes in the contents of B_2_O_3_, CaO, and Na_2_O increased by about 15% up to 150 ºC, they reached 34%–35% in the range of 150–400 °C compared with the initial values. In general, the amount of B_2_O_3_ increased by about 35% as a result of dehydration, while the amount of CaO and Na_2_O increased by about 34%. Thus, it is possible to increase the content of ulexite ores by dehydration. It is seen by examining these increases in Figure 8 that the amount of B_2_O_3_ reaches the maximum in 1 h at 5 g of ulexite ore and the minimum in 4 h at 1 g of ulexite ore. Similarly, with Na_2_O, while the maximum yield was reached at 1 g of ulexite ore in 1 and 5 h, the minimum yield was observed at 1 g of ulexite ore in 4 and 6 h. When examining the CaO yield, it is clear that the maximum yield is achieved at 5 g of ulexite ore and in 1 h, while the minimum yield is achieved at 1 g of ore in 4 and 6 h.

As a result of the experiments, a detailed table was prepared with the data obtained between the dehydration time and temperature considered effective in heat treatments and the amount of ore. With this table, different experiments were carried out and the changes of H_2_O, B_2_O_3_, Na_2_O, and CaO obtained with the studied parameters were determined as variables. One of the most important variables is the dehydration temperature. Other parameters are ore quantity and drying time. Only −325 mesh was used as the grain size. The importance of the input parameters used for the modeling is given in Table 3 and the summary table of the results is given in Table 4.

**Figure 9:**
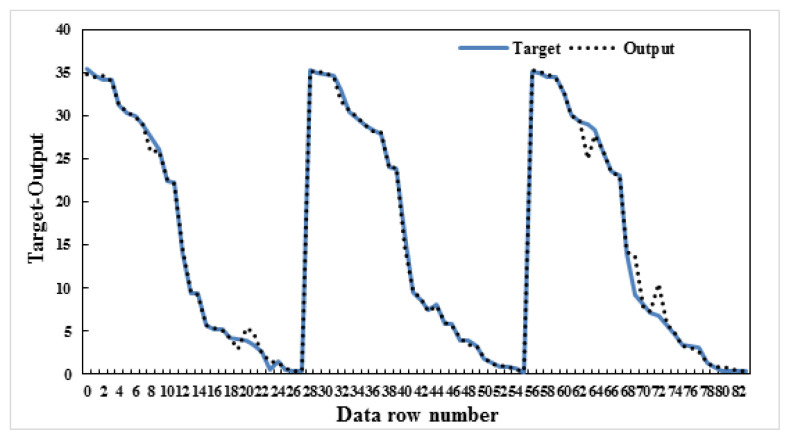
Actual and predicted values graph-H_2_O-model:5-7-1.

**Figure 10:**
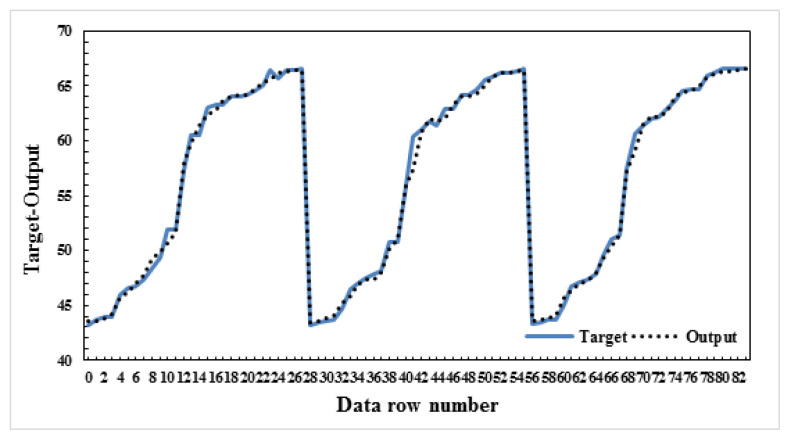
Actual and predicted values graph-B_2_O_3_-model:5-5-1.

**Figure 11:**
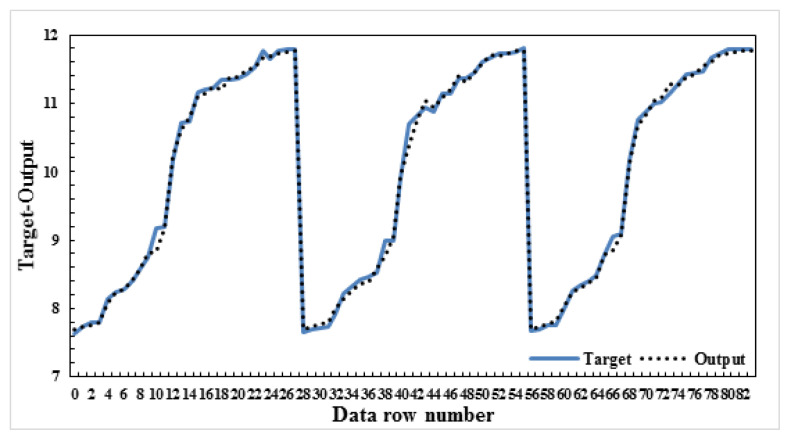
Actual and predicted values graph-Na_2_O-model:5-8-1.

**Figure 12:**
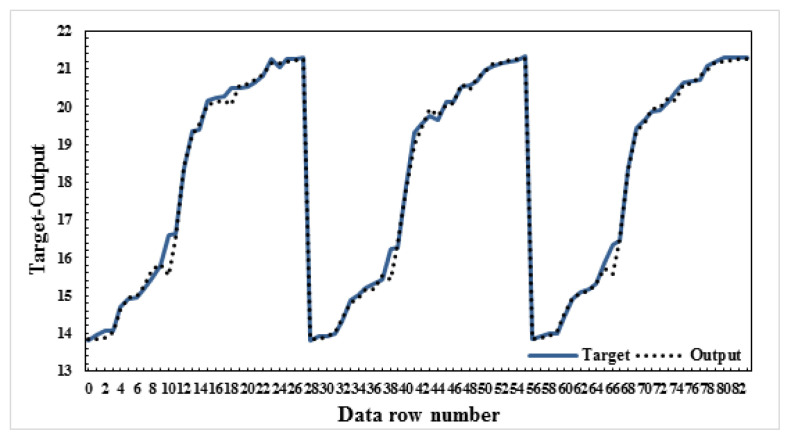
Actual and predicted values graph-CaO-model:5-6-1.

In this study, four different models were created by calculating the maximum water loss and the changes in B_2_O_3_, Na_2_O, and H_2_O yields of the ulexite ore subjected to dehydration. While creating the model, dehydration time and temperature, and ore amounts were used as parameter values. The significance levels of these parameters were determined and ANN modeling was performed. Several models were built on B_2_O_3_, Na_2_O, CaO, and H_2_O yields in this ANN modeling. All of these models were found to be successful, but among them model-1, model-2, model-3, and model-4 were selected to be the most successful four models. As mentioned above, the data resulting from the dehydration processes of ulexite ores were estimated using ANN models and the best result was obtained with the ANN model 5-8-1. It was found that there was a high correlation between the independent variables and the dependent variable. According to the correlation analysis, the most important parameters are the values of drainage temperature. As a result of the modeling, the graphs comparing the actual and the predicted values are shown in Figures 9–12.

In order to accurately predict at what temperature and time ulexite ores of different quality reach their maximum water losses and B_2_O_3_, Na_2_O, and CaO contents, the most effective parameters on the model must first be estimated.

As a result of the network structure determined by the 4 models created with the parameters used, the training of the network was performed using training algorithms. For the first model (5-5-1), the drainage temperature was determined to be the most important factor with a rate of 98.23%. For the second model (5-6-1), the most important factor was the dehydration temperature, which was determined to be 98.012%. For the third model (5-7-1), the most important factor was the dehydration temperature, which was determined to be 98.08%. For the fourth model (5-8-1), the most important factor was determined to be the dehydration temperature, which had a rate of 98.31%, and a training algorithm was created based on these results.

In this study, four different models were created and the values of grade changes and water loss rates in ulexite ores were estimated according to the demand situation. Based on the selected parameters, it was decided that the model with the lowest error (AE/ARE) and the highest R^2^ value would be the most appropriate model. As a result of the modeling, the average AE values were determined as follows: model-1: 0.34, model-2: 0.11, model-3: 0.61, and model-4: 0.06 (Table 4). As a result of the modeling, the average ARE values were determined as follows: model-1: 0.006, model-2: 0.006, model-3: 0.26, and model-4: 0.006 (Table 4). Likewise, as a result of the modeling, the average R^2^ values were determined as follows: model-1: 0.996, model-2: 0.995, model-3: 0.996, and model-4: 0.997 (Table 4). According to these results, the highest R^2^, lowest AE and ARE values were found in model-4 as 0.9974, 0.06, and 0.006, respectively. For the estimation of the dehydration process, an ANN model was created for each model and run several times, and model-4:5-8-1 was determined to be the best model among the models in this study. Considering the minimum error rates and the highest R^2^ values, it was found that model 5-8-1 provided the most accurate prediction values. The other models proposed in the study gave very close values to those of model-4. These models were chosen to predict the water content, the amount of ore with the contents of B_2_O_3,_ Na_2_O and CaO to be produced, temperature values and length of the time period without the need for experimentation in all subsequent dehydration processes. It was found when considered the proposed ANN models that the independent variables explained the dependent variable very effectively which means that the models are very successful.

The applications of boron ores may vary because of their crystal water content, which requires heat treatment depending on their uses. It is important to either reduce or completely remove this type of water, which is chemically bound in boron ores. Thanks to the heat treatment, the amount of B_2_O_3_ in the ore can be significantly increased. This ensures that boron ores achieve the desired quality for industrial applications [38]. As a result, both the additional enrichment cost is reduced and the selling price is increased, making a significant contribution to the economy. On the other hand, chemically bound water and other impurities in boron minerals increase transportation and energy costs in boric acid production. It is also possible to reduce the raw material to be processed by removing the water in the mineral. Therefore, boric acid producers prefer (chemically bonded) dehydrated boron ores. In future studies, new models can be created using genetic algorithms or hybrid methods [39]. Using these models, it was also shown that operations can be performed with less experimental effort in the same process.

## 4. Conclusion

It was found in this study carried out for the dehydration processes of ulexite ore that in dehydration above 450 °C, the solubility decreases with increasing temperature and the run-of-the-mill ulexite ores should be calcined at a temperature range of 350–450 °C, above which a process is not necessary. In the dehydration processes performed on run-of-the-mill ulexite ores for use in industrial applications, the B_2_O_3_ content increased by 50%–54% in the temperature ranges of 350–450 °C. The % Na_2_O, and % CaO contents also increased at the same rate.

In the evaluations made on the basis of R^2^ values in ANN models, it is seen that all of the models are successful. In general, R^2^ values of 0.90 and above are considered successful in ANN models. The R^2^ values of the four models selected in this study were also found to be 0.99 and above. If an evaluation is made within the four models; the highest R^2^ value was found to be 0.9974 for model-4 and this value was determined to be higher than the other three models. However, the R^2^ values of model-1, model-2, and model-3 are 0.9964, 0.9952, and 0.9958, respectively. As can be seen from these values, all the proposed models were successful. In examining the estimates obtained as a result of the studies from ANN, the dehydration time and temperature values could be estimated to obtain a product suitable for the desired grade and water content in ulexite ores.

This will avoid additional dehydration/calcination costs, reduce production costs, and save transportation and energy costs. This situation is expected to bring many advantages, especially in terms of marketing. This is because chemically bound water and other impurities in boron minerals increase transportation and energy costs in boric acid production. For this reason, boric acid producers primarily prefer “anhydrous” boron ores. Therefore, in this study, it was found that the results obtained in dehydration or calcination can be predicted by modeling ANN. In this way, it was shown that the additional cost of enrichment can be reduced, selling prices can be increased, thus making a serious contribution to the economy, and a product of the desired quality in boron ores can be obtained in a very short time.

## Figures and Tables

**Table 1:** Analysis results of ulexite ore.

Components	%
B_2_O_3_	43.1
CaO	13.8
Na_2_O	7.6
H_2_O	35.4

**Table 2:** Parameters used while creating ANN models.

Parameters	Parameter values	The reason for using this variable
**(C) (dehydration temperature), (ºC)**	50-100-150-200-250-300-350-400-450-500-550-600-650-700	To find the max water removal temperature, and the amounts of B_2_O_3_, Na_2_O, and CaO.
**((N) dehydration time (h))**	1-2-4-6	To find the most ideal and maximum time for water removal and B_2_O_3_, Na_2_O, and CaO amounts.
**((N) amount of ore (g))**	1-3-5	To be able to see the dehydration behavior and B_2_O_3_, Na_2_O, and CaO change in the increase in the amount of ore against the same grade.

**Table 3:** Significance of input parameters.

Input column name	Importance-(H_2_O), %	Importance-(B_2_O_3_), %	Importance-(Na_2_O), %	Importance-(CaO), %
Sample weight (g)	0.28	0.31	0.19	0.21
Dehydration temperature (ºC)	98.08	98.23	98.31	98.02
Dehydration time (h)	1.65	1.46	1.48	1.77

**Table 4:** Summary table of results.

Model-1: B_2_O_3_	Target	Output	AE	ARE
**Mean**	56.44	56.35	0.34	0.006
**SD**	8.87	8.80	0.41	0.007
**Min**	4.14	43.46	0.02	0.0004
**Max**	66.62	66.54	3.11	0.052
**Correlation**	**0.9983**			
**R-squared**	**0.9964**			
**Model-2: CaO**	**Target**	**Output**	**AE**	**ARE**
**Mean**	18.06	18.01	0.11	0.007
**SD**	2.84	2.85	0.16	0.01
**Min**	13.8	13.83	0.002	0.0001
**Max**	21.32	21.28	1.04	0.06
**Correlation**	**0.9978**			
**R-squared**	**0.9952**			
**Model-3: H** **_2_** **O**	**Target**	**Output**	**AE**	**ARE**
**Mean**	15.51	15.53	0.61	0.28
**SD**	13.28	13.13	0.59	0.61
**Min**	0.26	0.67	0.01	0.0006
**Max**	35.42	34.56	3.42	3.10
**Correlation**	**0.9980**			
**R-squared**	**0.9958**			
**Model-4: Na** **_2_** **O**	**Target**	**Output**	**AE**	**ARE**
**Mean**	10.00	9.98	0.06	0.006
**SD**	1.57	1.57	0.06	0.006
**Min**	7.64	7.70	0.0012	0.0001
**Max**	11.8	11.78	0.36	0.04
**Correlation**	**0.9987**			
**R-squared**	**0.9974**			
